# Secretory IgA: Designed for Anti-Microbial Defense

**DOI:** 10.3389/fimmu.2013.00222

**Published:** 2013-08-06

**Authors:** Per Brandtzaeg

**Affiliations:** ^1^Laboratory for Immunohistochemistry and Immunopathology (LIIPAT), Centre for Immune Regulation (CIR), University of Oslo, Oslo, Norway; ^2^Department of Pathology, Oslo University Hospital Rikshospitalet, Oslo, Norway

**Keywords:** mucosa, antibodies, commensals, pathogens, MALT, GALT, NALT, germinal centers

## Abstract

Prevention of infections by vaccination remains a compelling goal to improve public health. Mucosal vaccines would make immunization procedures easier, be better suited for mass administration, and most efficiently induce immune exclusion – a term coined for non-inflammatory antibody shielding of internal body surfaces, mediated principally by secretory immunoglobulin A (SIgA). The exported antibodies are polymeric, mainly IgA dimers (pIgA), produced by local plasma cells (PCs) stimulated by antigens that target the mucose. SIgA was early shown to be complexed with an epithelial glycoprotein – the secretory component (SC). A common SC-dependent transport mechanism for pIgA and pentameric IgM was then proposed, implying that membrane SC acts as a receptor, now usually called the polymeric Ig receptor (pIgR). From the basolateral surface, pIg-pIgR complexes are taken up by endocytosis and then extruded into the lumen after apical cleavage of the receptor – bound SC having stabilizing and innate functions in the secretory antibodies. Mice deficient for pIgR show that this is the only receptor responsible for epithelial export of IgA and IgM. These knockout mice show a variety of defects in their mucosal defense and changes in their intestinal microbiota. In the gut, induction of B-cells occurs in gut-associated lymphoid tissue, particularly the Peyer’s patches and isolated lymphoid follicles, but also in mesenteric lymph nodes. PC differentiation is accomplished in the lamina propria to which the activated memory/effector B-cells home. The airways also receive such cells from nasopharynx-associated lymphoid tissue but by different homing receptors. This compartmentalization is a challenge for mucosal vaccination, as are the mechanisms used by the mucosal immune system to discriminate between commensal symbionts (mutualism), pathobionts, and overt pathogens (elimination).

## Introduction

The existence of an external antibody system was proposed by Alexandre Besredka at the Pasteur Institute, Paris, when he in 1919 showed that rabbits, after oral immunization with killed Shigella, were protected against fatal dysentery irrespective of the serum antibody titer (1). Over the last 20 years before his death in 1940, he devoted most of his time to the study of mucosal immunization. In 1922 Arthur Davies, through his work as a physician for the British troops in Egypt, supported Besredka’s idea of a separate mucosal immune system by detecting antibodies against the dysentery bacillus in stools several days before such antibodies appeared in serum of infected patients (2). These and other pioneering studies on secretory antibodies have been discussed by Besredka (3) and Pierce (4).

A molecular basis for this field emerged when it was shown that saliva contains immunoglobulin (Ig) molecules (5). Conclusive evidence was not obtained, however, until the identification of different Ig classes was possible, and several laboratories reported that IgA predominates in most external secretions (6). The discovery by Thomas B. Tomasi and colleagues in USA, showing that secretory immunoglobulin A (SIgA) exhibits unique molecular properties, further intensified investigation of mucosal immunity (7). SIgA was found to be polymeric (mainly dimers) and covalently associated with an 80-kDa epithelial glycoprotein initially called “transport piece” and later named “secretory component” (SC). It was furthermore reported by Joseph F. Hereman’s laboratory in Belgium that the Ig class distribution of plasma cells (PCs) in the human gut differs strikingly from that in lymph nodes and bone marrow (8); in normal mucosal tissues, IgA^+^ PCs, and their immediate precursors (plasmablasts) are ∼20 times as numerous as IgG^+^ PCs.

**Figure 1:**
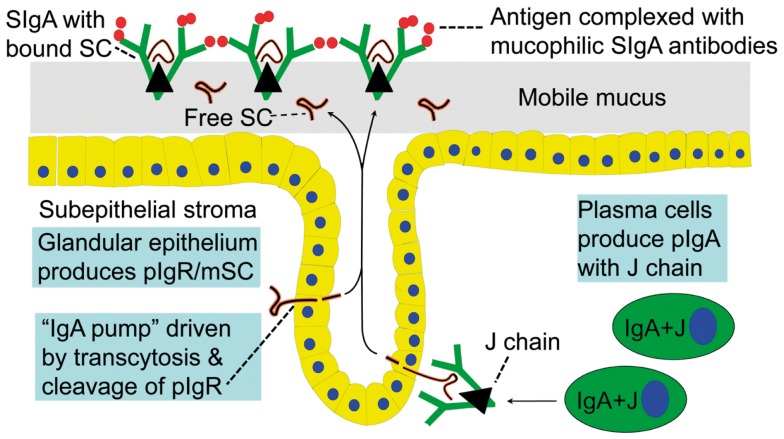
**Receptor-mediated epithelial export of polymeric IgA (pIgA, mainly dimers) to provide secretory IgA (SIgA) antibodies**. At the mucosal surface, SIgA antibodies together with mucus perform immune exclusion of antigens. The epithelial polymeric Ig receptor (pIgR) is expressed basolaterally, mainly in the intestinal crypts (glands), as membrane secretory component (mSC) and mediates external transcytosis of pIgA (and pentameric IgM, not shown). SIgA is released to the lumen with bound SC by apical cleavage of pIgR, in the same manner as unoccupied pIgR (carrying no ligand) is cleaved to provide free SC. Mucosal plasma cells produce abundantly pIgA with incorporated J chain (IgA + J), which is required for high-affinity epithelial binding of the pIgR ligands. Modified from Brandtzaeg (16).

In 1973, our laboratory provided the first direct evidence that human mucosal IgA^+^ PCs produce mainly dimers and perhaps some larger polymers (collectively called polymeric, mainly IgA dimers, pIgA) rather than monomers (9), and in 1974 this characteristic was found to be associated with co-expression of a 15-kDa disulfide-linked polypeptide called joining (J) chain (10). In the late 1960s we had observed that not only pIgA but also pentamers of IgM are preferentially transferred to external secretions such as saliva, apparently because of a common epithelial transport system (11, 12). Secretory IgM (SIgM) in parotid saliva was subsequently shown to be only non-covalently associated with SC (13), but in the gut epithelium IgM was found by immunoelectron microscopy to follow the same intracellular vesicular transfer route as pIgA and SC, while the secretory epithelial cells apparently were devoid of IgG (14). A shared receptor-mediated mechanism involving endocytosis and transcytosis therefore seemed to exist for SIgA and SIgM formation (9, 10, 15–17). Our transport model was based on a suggested crucial cooperation between J-chain-expressing mucosal IgA^+^ (and IgM^+^) PCs and SC-expressing serous-type of secretory epithelial cells (Figure 1).

## Export of Secretory Antibodies

Membrane SC is a carbohydrate-rich glycoprotein of ∼100 kDa constitutively expressed basolaterally on secretory epithelial cells (Figure 1), where it exhibits strong non-covalent affinity for J-chain-containing pIgA and pentameric IgM (18). It belongs to the Ig supergene family with five extracellular domains and is now usually referred to as the polymeric Ig receptor (pIgR). Its human gene has been cloned and characterized (19), and several DNA elements could explain its remarkably high constitutive and cytokine-enhanced expression (20). Interferon-γ (IFN-γ) was the first cytokine shown to increase epithelial pIgR/SC expression and it was taken to be particularly responsible for the enhanced pIg export seen in concert with an intensified local immune response (21). IFN-γ-responsive DNA element in the upstream promoter and exon 1 of the pIgR gene have been identified (22), but there are also elements responsive to regulatory factors in the first intron (20). Altogether, both steroid hormones and proinflammatory cytokines can upregulate pIgR, including interleukin (IL)-17 which is particularly abundant at mucosal sites (23). Microbial components interacting with epithelial pattern recognition receptors (PRRs), such as Toll-like receptors (TLRs), can do the same (24).

**Figure 2:**
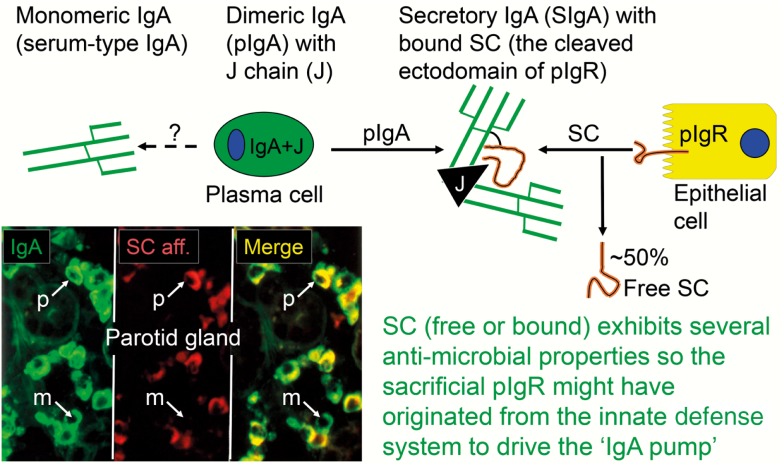
**Generation of secretory IgA (SIgA) and free secretory component (SC)**. SIgA is formed as a hybrid antibody molecule stabilized by a disulfide bridge between the two cell products. The amount of dimeric IgA (pIgA) produced by a plasma cell depends on its level of J-chain expression, which generally is high in mucosal and glandular tissue. Inset (left) shows direct demonstration of abundant cytoplasmic expression of pIgA (p) in most plasmablasts and plasma cells in the parotid gland achieved by *in vitro* affinity test with free SC on tissue section as described (9), whereas a single cell producing only monomers (m) is seen in this field. On average, ∼50% of SC occurring in various secretions is in a free form (unoccupied by ligand). The immunostained panel is from Brandtzaeg (45).

When pIgR reaches the apical surface of the epithelial cell, SIgA and SIgM are exocytosed after cleavage of the receptor; only its C-terminal segment remains for intracellular degradation (Figure 1). The extracellular part of pIgR (∼80 kDa) is exceptionally carbohydrate-rich (25), and when incorporated into the SIg molecules as bound SC it endows particularly SIgA (where it becomes disulfide-linked) with resistance against proteolytic degradation (26). Excess of unoccupied pIgR is released in the same manner by proteolytic cleavage to form so-called free SC (Figures 1 and 2) according to the internationally recommended nomenclature (27). This 80-kDa glycoprotein can be found in most exocrine secretions (12), and on average ∼50% of the exported SC occurs in a free form (28). This “sacrificial” nature of pIgR explains the need for its high level of constitutive expression (20). Importantly, both free SC and bound SC show several innate immune functions such as inhibition of epithelial adhesion of certain Gram-negative bacteria and neutralization of bacterial toxins (26). By equilibrium with bound SC, free SC in secretions also exerts a stabilizing effect on the quaternary structure of SIgM in which bound SC is only non-covalently linked (13).

The binding sites of pIgA and pentameric IgM initially contacting the first extracellular domain of pIgR have largely been defined (29). In addition, it has been shown that the J chain is crucial for the initial non-covalent complexing and stabilization between the Ig polymers and pIgR (or free SC) in *in vitro* experiments (18, 30). Thus, our original proposal that the J chain and pIgR/SC are involved in a “lock and key” mechanism in the selective epithelial export of pIgA and pentameric IgM, is now firmly established (31–33). The J chain is normally produced preferentially by mucosal PCs (34), perhaps reflecting a recent generation of their precursors in germinal centers (GCs) of mucosa-associated lymphoid tissue (MALT), while little or no J-chain expression would signify several precursor rounds through GCs according to the “decreasing potential” hypothesis (35). However, the J chain can only become disulfide-linked to the Fc regions of IgA and IgM which carry a small tailpiece in their heavy (H) chains (36). When it is produced by other PC classes (Table 1), it therefore remains in a free form and is degraded intracellularly without being released from the cells in detectable amounts (37, 38). That most PCs at a normal secretory effector site contain pIgA with incorporated J chain, is demonstrated by the fact that the cytoplasm of these cells in a tissue section binds free SC when it is added *in vitro* (Figure 2).

**Table 1:** **J-chain positivity (%) of mucosal plasmablasts and plasma cells**.

Exocrine tissue site	Ig class expression			
	IgA	IgM	IgG	IgD
Mammary glands	94	100	56	100
Salivary and lacrimal glands	92	100	44 (72)*	95
Normal nasal mucosa	98	100	69	100
Normal small intestinal mucosa	99	100	87	ND

*Data from IgA-deficient individuals.

*ND, not determined*.

*Based on published data from the author’s laboratory*.

**Figure 3:**
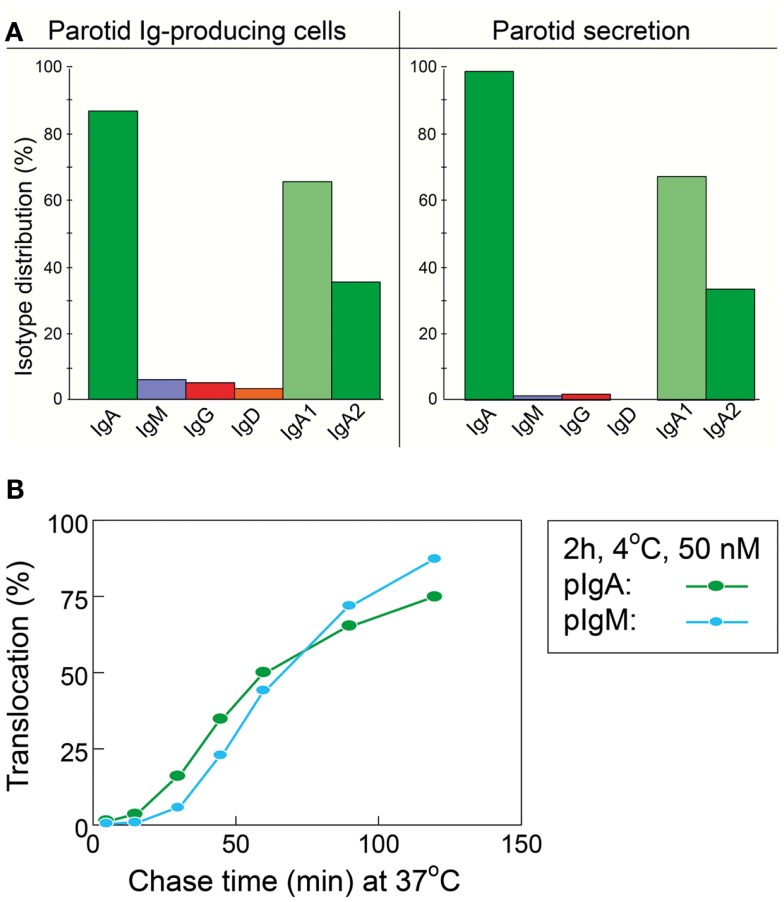
**Relationship between local production of Ig isotypes by gland-associated plasma cells and Ig transfer by secretory epithelium**. **(A)** Compared with the local production in the parotid gland, export of IgA into stimulated secretion is clearly favored over export of IgM (and IgG and IgD), whereas translocation of the two subclasses of IgA appears to handled equally well by the glandular epithelium. **(B)** Comparison of epithelial translocation of dimeric IgA (pIgA) and pentameric IgM (pIgM) was performed *in vitro* with polarized MDCK cells transfected with the human polymeric Ig receptor. Cells were incubated with ^125^I-labeled pIgA or pIgM for 2 h at 4°C, washed for 10 min at 4°C, and chased at 37°C for different times as indicated. Translocation is expressed as the cumulative appearance of ^125^I-pIgA and ^125^I-pIgM in the apical medium. Each point represents mean result of three filters for pIgA and pIgM translocation at 50 nM ligand concentration. Adapted from Norderhaug et al. (29).

As mentioned previously, SIgM is not secondarily stabilized by bound SC through disulfide bonding, and its resistance to proteolytic degradation is inferior compared to SIgA. Also, when comparing the proportions of parotid PC classes and the IgA-to-IgM concentration ratio in the secretion (Figure 3A), the glandular export of pIgA is favored over that of pentameric IgM by a factor of ∼5 (or 12-fold on a molar basis) (39). This is not explained by different handling of the two polymers by pIgR (Figure 3B) but is due to diffusion restriction for the relatively large IgM pentamers through stromal matrix and basement membranes, inhibiting its access to the basolaterally expressed pIgR. In fact, human pentameric IgM shows much higher affinity for free SC *in vitro* than does pIgA (30).

Of the two subclasses of IgA, IgA2 is more stable than IgA1 because the short hinge region renders it resistant to certain bacterial proteases (40). Molecular modeling suggests that its short hinge also explains a rigid and non-planar structure which facilitates better multivalent binding of IgA2 to antigens on bacterial surfaces (41). Therefore, it is interesting that a large proportion (40–60%) of the IgA^+^ PCs in distal gut mucosa produce IgA2 (42, 43). In this respect, the salivary glands are intermediate between the upper airways and the distal gut, a disparity that clearly reflects regional immunoregulatory differences (34). In agreement with the similar affinity of IgA1 and IgA2 for free SC *in vitro* (30), however, both subclasses appear to be equally well exported by pIgR into the external secretions (Figure 3A), such as parotid saliva (44).

As alluded to previously (Figure 2), evaluation of IgA^+^ PCs at secretory effector sites for J-chain expression and *in vitro* cytoplasmic affinity for free SC (marker of pIgA production), has indicated that almost 90% of them are variably involved in production of polymers (45). These pIgA molecules are immediately available for the unique pIgR-driven epithelial transport system which generates both free SC and the hybrid SIgA molecule (Figures 1 and 2), where the bound SC in a changed conformational shape covers most of the J chain, according to recent modeling studies (41, 46). Thus, the many cartoons in the literature depicting bound SC wrapped around the Fc portions of the two IgA subunits, are definitely wrong.

There have been many conflicting opinions about the epithelial elements expressing pIgR/SC. In our laboratory, this receptor has been localized by immunostaining mainly to the serous-type of epithelial cells, regardless of the mucosal or glandular body site investigated (47–49). But it remains unknown how locally produced pIgA and pentameric IgM molecules are directed in their diffusion through the stromal ground substance to reach receptor-expressing epithelial elements instead of being drained by lymph to the blood circulation (17). Thus, there is normally no enrichment of pIgA in thoracic duct lymph and portal vein blood (50).

## Defense Mechanisms of Secretory Antibodies

The chief defense function of SIgA appears to be binding of soluble or particulate antigens in the action referred to as immune exclusion (51). The remarkable stability of SIgA makes it well suited to function in protease-containing secretions, and its enzymatic resistance seems to be enhanced when the antibody is complexed with antigen (52). Notably, certain bacteria such as *Neisseria gonorrhoea*, *N. meningitidis*, *Streptococcus pneumoniae*, and *Hemophilus influenzae* produce enzymes that can selectively cleave SIgA1 in its extended (13-amino acid) hinge region. The same is true for oral bacteria, especially some strains of *S. sanguinis* (previously *S. sanguis*) and *S. mitior* (previously *S. mitis*) but also *Porphyromonas/Prevotella* (previously *Bacteroides*) and *Capnocytophaga* species which are involved in periodontal disease (40). On average, at least 60% of salivary IgA consists of the IgA1 isotype (Figure 3A), and parotid antibodies to *S. mutans* occur predominantly in this subclass, whereas reactivity to lipoteichoic acid from *S. pyogenes* and to lipopolysaccharides (LPS) from *Porphyromonas gingivalis* (previously *Bacteroides gingivalis*), *Bacteroides fragilis*, and *Escherichia coli* is carried mainly by in the IgA2 isotype (53).

Although Fabα fragments released by the IgA1 proteases may retain antigen-binding capacity (54), this immune reaction may be adverse rather than protective. Such fragments may shield microorganisms from the defense function of SIgA antibodies and may even enhance epithelial colonization and thereby promote invasiveness (55), whereas intact SIgA can specifically inhibit cellular attachment and penetration of for instance influenza virus, in contrast to monomeric IgA or IgG neutralizing antibodies (56). *In vivo* coating of bacteria with IgA can be directly demonstrated by immunostaining; and although this apparently does not inhibit bacterial growth (57), it is considered to provide containment of the microbiota and counteract invasiveness (Table 2). But bacterial IgA coating is no proof of antibody reactivity because many strains of group A or B streptococci possess Fcα receptors (58). Nevertheless, binding of SIgA to bacteria via Fc interactions may be of similar functional importance as Fab-mediated antibody coating, and the same regards microbial interactions with glycans of bound SC in SIgA (26). By affinity for mucin SIgA may also be involved in biofilm formation (59), and it was recently reported that mannose-containing oligosaccharides within human SIgA can alter the virulence phenotype of *Vibrio cholerae* such as biofilm formation (60).

**Table 2:** **Anti-microbial effects of secretory IgA antibodies**.

SIgA is dimeric/polymeric and exhibits T-shaped Fab fragments, therefore exerting efficient microbial agglutination and virus neutralization
SIgA performs non-inflammatory extracellular and intracellular immune exclusion by inhibiting epithelial adherence and invasion
SIgA exhibits cross-reactive (“innate-like”) activity as well as high-affinity somatic mutants and provides cross-protection in a partially vaccinated population (“herd protection”)
SIgA (particularly SIgA2) is quite stable (bound secretory component stabilizes both subclasses)
SIgA is endowed with mucophilic and lectin-binding properties (via bound secretory component in both subclasses and mannose in IgA2)

*Modified from Brandtzaeg (51)*.

**Figure 4:**
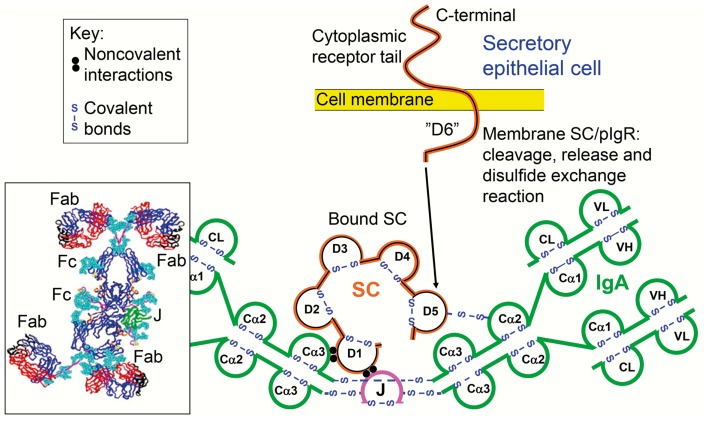
**Synopses of the structural basis for the excellent anti-microbial binding properties of secretory IgA (SIgA)**. Domain interactions in the formation of SIgA based on data reviewed in Norderhaug et al. (29). Non-covalent interactions are shown between the J chain (J) and the extracellular domain 1 (D1) of bound secretory component (SC), and covalent disulfide bonding is indicated between cysteine 467 or 502 in D5 of bound SC and cysteine 311 in the Cα2 domain of one of the two IgA subunits. Some studies have indicated that there may be two J chains in dimeric IgA (30). Insert to the lower left is from modeling data for dimeric IgA1 based on X-ray and neutron scattering in solutions published in Bonner et al. (63). Note the T-shape of the Fab fragments, allowing for antibody binding to large particles like bacteria. V, variable region; C, constant region; L, light chain; H, heavy chain; Fab, fragment antigen binding; Fc, fragment crystallizable.

Many identified strategies may contribute to SIgA-mediated immune exclusion of antigens (Table 2). In addition to more efficient antigen binding, complexing, and neutralization (61), SIgA antibodies show better agglutinating properties than monomeric IgA, which may be partly explained by interactions with mucin (62). The combined effect of the dimeric structure and the T-shaped Fab fragments (63) – allowing SIgA antibodies to grasp big particulate antigens such as bacteria – can largely explain the superior biological properties of SIgA antibodies (Figure 4). Their function appears to be further amplified by a high level of polyreactivity or cross-reactivity (64), as discussed later. Finally, in complex with antigen the affinity of SIgA2 for as yet unknown receptor(s) on epithelial M cells of gut-associated lymphoid tissue (GALT) is enhanced (52), thus promoting mucosal immunity against targeted antigens by “immune inclusion” which may be particularly important in immunological integration between the mother and her breastfed baby (65).

An interesting example of the potential of SIgA-mediated immune exclusion is the experimental results obtained with mucosal immunization against *Mycobacterium tuberculosis* (66, 67). The fight against tuberculosis requires new thinking and innovative approaches, which are central to developing novel active or passive immunization strategies. The possible role of SIgA in this respect has been shown in an infection model and intranasal vaccination in pIgR knockout mice which are deficient in secretory immunity (68, 69), and by nasal administration of – or bacterial preincubation with – purified colostral SIgA from unimmunized (naïve) women in an *M. tuberculosis*-based murine model (70).

## Inductive Sites for Mucosal B-Cell Activation

**Figure 5:**
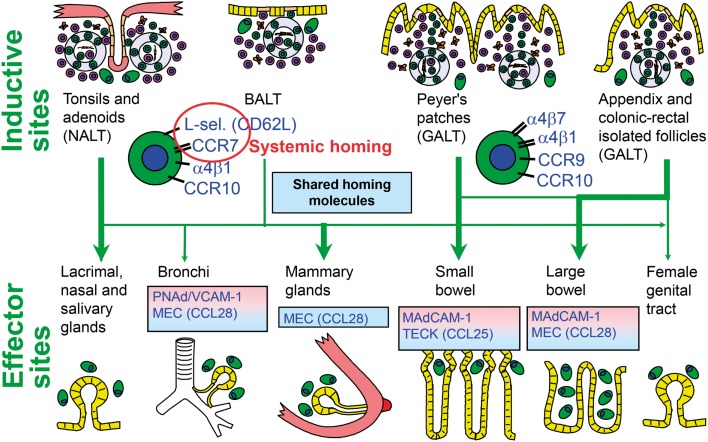
**Homing properties of human mucosal memory/effector B cells**. Putative scheme for compartmentalized migration of B cells from inductive (top) to effector (bottom) sites. Depicted are more or less preferred pathways (graded arrows) presumably followed by mucosal B cells activated in nasopharynx-associated lymphoid tissue (NALT) represented by palatine tonsils and adenoids, bronchus-associated lymphoid tissue (BALT), and gut-associated lymphoid tissue (GALT) represented by Peyer’s patches, appendix, and colonic-rectal isolated lymphoid follicles. The principal homing receptor profiles of the respective B-cell populations, and adhesion/chemokine cues directing extravasation at different effector sites, are indicated (pink and blue panels) – those operating in lactating mammary glands apparently being shared for NALT- and GALT-derived cells. Homing molecules integrating airway immunity with systemic immunity are encircled in red. Adapted from Brandtzaeg (51). MEC, mucosae-associated epithelial chemokine; TECK, thymus-expressed chemokine.

Initial immune stimulation to generate memory/effector B cells for mucosal pIgA responses takes place mainly in MALT structures, particularly Peyer’s patches (PPs) of the distal ileum and other parts of GALT, such as the numerous isolated lymphoid follicles (ILFs) and the appendix (71). From these inductive sites the activated B-cells reach peripheral blood by migrating through lymph and draining lymph nodes and subsequently extravasate at, or are excluded from, secretory effector sites on a competitive basis – depending on complementary adhesion molecules and chemokine–chemokine receptor pairs (71, 72). The homing is successfully accomplished when it is directed by interactions between several dynamically regulated endothelial adhesion molecules or “addressins” and the corresponding ligands (“homing receptors”) expressed on the memory/effector cells (Figure 5). By such complex mechanisms, mucous membranes and exocrine glands are furnished with locally produced antibodies, partly in an integrated way ensuring a variety of specificities at every secretory site, but also in a compartmentalized manner making the secretory immune system less “common” than previously believed (27, 34, 71).

It is not always well delineated which MALT structures are most important for induction of SIgA responses subsequently expressed at a specific secretory effector site, but there is convincing evidence both in animals and humans that activated B-cells migrate from GALT to the upper aerodigestive tract and lactating mammary glands (73–76). However, recent studies point to the possibility that nasopharynx-associated lymphoid tissue (NALT), such as the unpaired nasopharyngeal tonsil (often called adenoids) and the paired palatine tonsils in humans, may be most important as an inductive site for mucosal memory/effector B cells in this region (77, 78). Although it remains uncertain to what extent these lymphoepithelial structures of Waldeyer’s ring are functionally comparable to rodent NALT (79), they are strategically located to orchestrate mucosal immunity against both airborne and alimentary antigens. Moreover, they are well designed for immune induction because of their deep and branched antigen-retaining crypts and the absence of antigen-degrading digestive enzymes.

There is, moreover, circumstantial evidence to imply that the human GALT system differs considerably from that of NALT with regard to B-cell precursor differentiation and/or immunoregulatory events involved in this process. First, a striking disparity exists between the two regions in terms of IgD responses, particularly in IgA deficiency (34). Second, there is also a disparity in the subclass distribution of IgA^+^ cells, as mentioned previously. Although some studies have concluded that the predominance of IgA2^+^ plasmablasts/PCs in the distal intestine (particularly in the colon) can be explained by *in situ* switching to this subclass in the lamina propria (80), we have found by molecular methods that such a contribution must be negligible or absent; the IgA-subclass profile seems, instead, to be imprinted in MALT structures before the homing of activated B cells to secretory effector sites takes place (Lin et al., in revision).

## Homing Molecules Guiding the Migration of Mucosal B Cells

In an *S. mutans*-based caries model in rats, intranasal immunization with a recombinant bacterial fusion protein induced salivary IgA antibodies and serum IgG and IgA antibodies (81). Also, direct immunization of human palatine tonsils, and particularly nasal vaccination, gave rise to local B-cell responses in tonsils and adenoids as well as circulating specific B cells which were excluded from the intestinal mucosa (82). Similarly, infants dying of sudden infant death syndrome (SIDS) were found to have overstimulated tonsillar GCs reflected by an increased number of IgG^+^ and IgA^+^ PCs, probably caused by airway infection (83); and such activated B cells were apparently distributed in excessive numbers to regional secretory effector sites, including the parotid glands (84), thereby explaining the increased levels of salivary IgA and IgM in SIDS (85).

Evidence is thus accumulating to support the notion that human NALT supplies secretory effector sites of the upper aerodigestive tract with activated pIgA^+^ precursor cells (78, 86). One reason for the suggested homing dichotomy between this region and the small intestine appears to be differences in the employed homing molecules (72). The leukocyte integrin α4β7 is important for B-cell extravasation into the gut lamina propria by interaction with the mucosal addressin cell adhesion molecule (MAdCAM)-1 expressed on the intestinal microvascular endothelium (Figure 5); but this integrin does not appear to be important for homing to the airways and salivary glands (34). Also the involved chemokine receptor–chemokine interactions (CCR9–CCL25 versus CCR10–CCL28) show a striking dichotomy between the two body regions (87). The low expression level of gut-homing molecules after NALT immunization, particularly α4β7, has been shown in mice to be the direct reason for exclusion of the activated B cells from small intestinal mucosa (72). Human NALT induction induces, instead, α4β1 interacting with vascular cell adhesion molecule (VCAM)-1 and also the expression of the systemic homing molecules l-selectin (CD62L) and CCR7 (78). This probably reflects that palatine tonsils and adenoids act as a “cross-road” between mucosal and systemic immunity (Figure 5).

Sublingual immunization in mice with stimulation of B cells in cervical lymph nodes apparently results in dissemination of immunity by the same homing molecules as those operating after NALT stimulation, but may be a safer approach with no possibility for redirecting antigens/adjuvants to the brain (88, 89). Sublingual instead of subcutaneous allergen administration is an established alternative approach to desensitize pollen-allergic patients, and such sublingual immunotherapy (SLIT) is now being tested also for food allergens. Interestingly, in a SLIT trial for peanut allergy, 6 of 10 patients showed significant induction of IgA (and SIgA) peanut-reactive antibodies in saliva (90).

Still there is a need for more extensive clinical studies, perhaps focusing collectively on the adenoids/palatine tonsils and cervical lymph nodes, in addition to GALT, as inductive lymphoid tissue for regional SIgA responses. For instance, some 20–30 years ago, reports relevant for human NALT suggested reduced salivary IgA levels in children with recurrent tonsillitis (91) or with adenoid hyperplasia (92). Decreased J-chain expression is a consequence of recurrent tonsillitis, and to a lesser extent adenoid hyperplasia, implying that chronic inflammation may compromise the potential of Waldeyer’s lymphoid ring to furnish the regional SIgA system with pIgA^+^ plasmablasts (78).

## Cellular and Molecular Interactions in Antibody Induction

Mucosa-associated lymphoid tissue structures are principally similar to lymph nodes but they are not encapsulated and have no afferent lymph supply. Antigens for immune stimulation therefore have to be taken up through a follicle-associated epithelium which is equipped with very thin M (“microfold” or membrane) cells which are specialized for sampling and transport of especially particulate antigens like bacteria (34).

### The germinal center reaction

Naïve T and B lymphocytes enter MALT structures through specialized high endothelial venules. The primary lymphoid follicles are aggregates of such recirculating B lymphocytes (sIgD^+^IgM^+^) which pass into a network formed by antigen-capturing follicular dendritic cells (FDCs). The origin of FDCs remains obscure, but both their development and the clustering that allows follicle formation depend on lymphotoxin (LT) signaling (93). Experimental evidence suggests that the B cells are one important LT source (94–96). Among the actions of the soluble homotrimer LTα, are augmentation of B-cell proliferation and expression of adhesion molecules, while mice deficient in transmembrane LTβ have no detectable FDCs (97).

**Figure 6:**
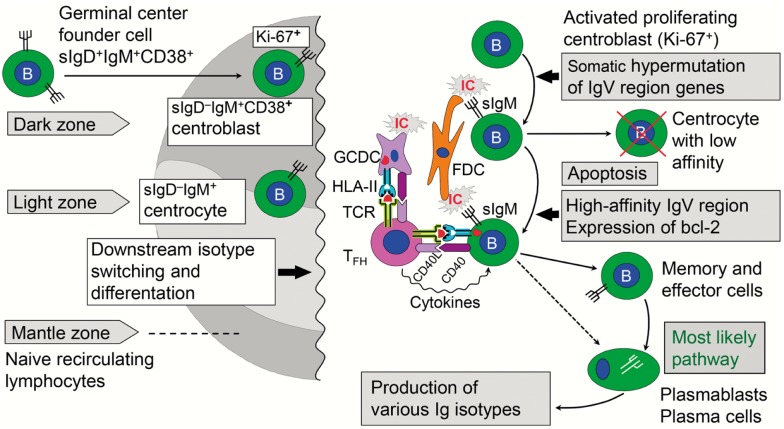
**Immune events taking place in the dark and light zones of human germinal center in secondary lymphoid follicle**. The “germinal center founder cells” receive their initial stimulation through cognate interaction with activated CD4^+^ helper T cells just outside the lymphoid follicle before they enter it to become proliferating centroblasts forming the dark zone. Affinity maturation of sIgM as part of the B-cell receptor is achieved after somatic hypermutation by competition for antigen presented in ICs on FDCs. Antigen taken up by the B cells is further presented in a cognate fashion to T_FH_ cells, which also receive stimulatory signals from GCDCs. The activated T_FH_ cells act on the B cells with their cytokines, thereby mediating expansion of high-affinity B-cell clones in the light zone. Further details are discussed in the text. sIg, surface immunoglobulin; bcl-2, anti-apoptotic B-cell lymphoma protein; IC, immune complex; FDC, follicular dendritic cell; GCDC, germinal center dendritic cell; HLA-II, HLA class II molecule; TCR, T cell receptor; T_FH_, follicular helper T cell; CD40L/CD40, costimulatory molecules.

**Figure 7:**
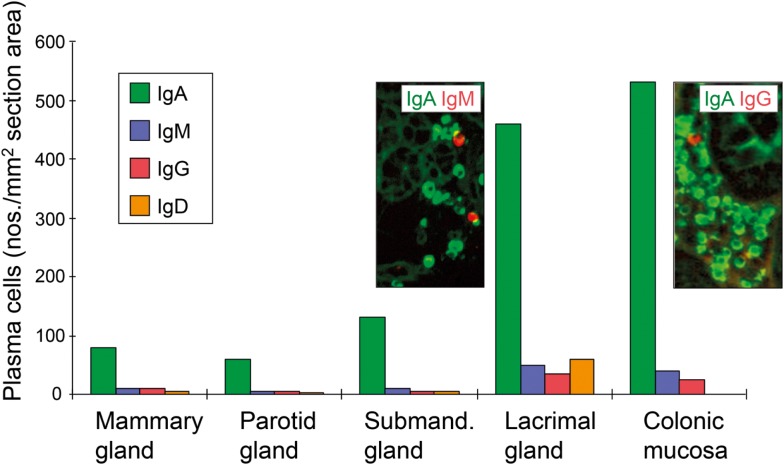
**Average tissue densities of plasmablasts/plasma cells producing different Ig classes at various normal secretory tissue sites as indicated**. Representative areas illustrating merge of immunofluorescence staining (see color key) are shown for submandibular gland and colonic lamina propria. Based on published data from the author’s laboratory.

The GC reaction turns the primary into a secondary follicle – polarized into a “dark zone” (dominated by proliferating centroblasts) and a “light zone” (dominated by centrocytes) where cells proceed to memory B-cell or terminal plasmablast/PC differentiation (Figure 6). In humans, this process has been extensively studied in tonsils (98), but much relevant mechanistic information relies on observations of lymph nodes and spleen from immunized animals (99). In general, GCs are of vital importance for T cell-dependent stimulation of conventional (B2) B cells, affinity maturation of B-cell receptor (BCR), and Ig-isotype switching. This means that the decision to make pIgA with co-expressed J chain has to take place in the GCs of MALT; but it remains elusive why PPs are superior in this respect (34) – as reflected in the high density of plasmablasts/PCs present in the intestinal lamina propria (Figure 7).

Naïve B cells that leave the primary follicles are first stimulated in the T cell zone just outside the follicle by cognate interactions with activated CD4^+^ T cells, which have been presented with processed antigen by MHC class II-expressing interdigitating DCs (100). In an interplay between the chemokine receptors CXCR5 and CCR7 and the G protein coupled receptor for EBV-induced molecule 2 (EBI2, also known as GPR183), further B-cell differentiation depends on the tissue niche where the cells end up – low-affinity extrafollicular PCs or high-affinity memory B cells and GC plasmablasts/PCs (101, 102). For the latter fate, there seems to be a BCR affinity-based pre-GC checkpoint operating in the cognate interaction between B and T cells taking place adjacent to the follicle (103). As described for human tonsils (104), those B cells that escape this selection re-enter the follicle and become proliferating sIgD^+^IgM^+^CD38^+^ GC “founder cells” forming the dark zone (Figure 6). A critical step allowing this decision seems to be downregulation of surface-expressed EBI2 (105).

The extrafollicular B cells are initially stimulated to produce unmutated IgM (and some IgG) antibody which binds circulating antigen with low affinity. Although still somewhat unclear, it seems that some of these cells use their complement receptors to carry opsonized antigen or immune complexes into the follicles to become deposited on FDCs (106). Antigen is retained in this network for prolonged periods to maintain B-cell memory (35). A role for IgM in the induction of secondary immune responses with antibody affinity maturation, has been strongly supported by observations in knockout mice lacking natural (“non-specific”) background IgM antibodies (107).

It remains elusive which factors govern the GC decision for the selected high-affinity memory/effector B cells to go into terminal differentiation to become plasmablasts/PCs (Figure 6); but it is known that the transcription factor paired box protein-5 (Pax5) represses genes associated with PC differentiation and function, while expression of B lymphocyte-induced maturation protein-1 (Blimp-1) is required for such development to take place (108). It is also clear that the complement receptors CR1/CR2 (CD35/CD21) play a crucial role in the GC reaction by acting as a signal-transducing complex. CD21 is expressed abundantly on both B cells and FDCs, and may thus function not only by localizing antigens to the FDCs but also by lowering the threshold of B-cell activation via recruitment of CD19 into the BCR (109).

Activation of complement on FDCs is controlled by regulatory factors when these cells retain immune complexes (Figure 6), but some release of inflammatory mediators may cause edema that facilitates dispersion of FDC-derived “immune complex-coated bodies,” or iccosomes, thereby enhancing the BCR-mediated uptake of their contained antigens by B cells with sufficient affinity (110, 111). The antigen will next be presented in a cognate fashion by these B cells to special follicular T helper (T_FH_) cells (112) which in this manner are stimulated to secrete cytokines – particularly IL-21 – driving the expansion of the selected high-affinity B-cells (Figure 6). The capacity of T_FH_ cells to secrete IgA-switch factors such as transforming growth factor (TGF)-β, and the local availability of the vitamin A metabolite retinoic acid, are probably important for the IgA-inducing capacity of a particular MALT structure (51, 71).

### Alternative induction and recruitment of mucosal B cells

Several components of the innate immune system, in addition to complement, may be involved in the GC activity, such as the cytokines A Proliferation-Inducing Ligand (APRIL) and B-cell-Activating Factor of the TNF Family (BAFF). Under certain conditions, IgA differentiation driven by gut bacteria may even bypass the usual sIgM (or sIgD)-BCR and T cell requirement (113) – that is, CD40–CD40L/CD154 interactions (Figure 6). Nevertheless, in mice experiments there always appears to be a dependency on some follicle-like aggregates of B cells (114) which, interestingly, may lack antigen-retaining FDCs and GCs (115, 116). Normally, however, the GC reaction and BCR affinity maturation are driven by competition for a limited amount of available antigen on FDCs, which may be governed via feedback from the pressure of antibodies produced by GC-derived plasmablasts/PCs (117). An additional innate drive may be particularly relevant for the numerous ILFs (71), and the B-cells resulting from mechanisms such as superantigen stimulation may survive with a restricted repertoire and rather low affinity (118).

In mice, the peritoneal cavity with its omentum is recognized as a non-MALT compartment where precursor cells for IgA production are generated (119), perhaps providing up to 50% of the intestinal IgA^+^ PCs (120). The precursors are self-renewing sIgM^+^ B1 (CD5^+^) cells, which can give rise to polyreactive (“natural”) SIgA antibodies, particularly directed against polysaccharide antigens from commensal bacteria as a result of T cell-independent responses (115). The substantial capacity of these B cells to produce IL-10 makes this leukocyte population an interesting candidate for immunoregulatory involvement (121). However, the relationship of murine B1 cells to the conventional bone marrow-derived B2 cells remains elusive. Notably, rather than being encoded only in germline, B1 cells from mice may show considerable somatic hypermutation (SHM) of Ig H-chain V-region (V_H_) genes as a sign of selection (122, 123).

It is controversial where the murine B1 subset differentiates to the IgA phenotype, although the intestinal lamina propria has been suggested as a class-switch site (124, 125). Notably, however, there is no evidence to suggest that peritoneal B1 cells contribute to mucosal IgA production in humans (126, 127), where a similar subset of B cells has not been conclusively defined. A minor subset of CD43^+^CD27^+^ B1-like human cells has recently been characterized in functional terms, but there is no indication that it contributes to mucosal immunity (128). Nevertheless, considerable levels of polyreactive or cross-reactive SIgA antibodies directed against self as well as microbial antigens occur in human external secretions (129). One reason for this could be bacterial T cell-independent polyclonal activation of GALT.

## B-Cell Fate at the Secretory Effector Site

B-cell homing to different effector sites seems to be antigen-independent, but topically available antigens may nevertheless contribute to local retention, proliferation, and differentiation of the extravasated cells. As reviewed elsewhere, experiments in gene-manipulated mice have suggested that signaling through LTβR on lamina propria stromal cells is necessary for the persistence of IgA^+^ plasmablasts/PCs in intestinal mucosa (34). In addition, PC retention and survival depend on a variety of cytokines and chemokines/chemokine receptors as well as adhesion molecules, including the extracellular matrix receptor CD44 expressed at high levels on post-GC B cells. However, the factors triggering terminal B-cell differentiation at secretory effector sites remain elusive, although IL-5, IL-6, and IL-10 may be particularly important. Notably, as evidenced in the multiple-intestinal-loop model in lambs, topical exposure to antigen has a marked impact on site-specific accumulation of specific IgA^+^ PCs, thereby influencing the observed B-cell homing pattern but without imposing any selectivity on the extravasation step of memory/effector cells. Thus, GALT-derived blasts have been shown to home to presumably antigen-free neonatal intestinal mucosa and to fetal gut grafted under the adult kidney capsule of experimental animals.

Antigen-driven proliferation of IgA^+^ B cells has been observed in intestinal lamina propria of experimental animals, especially in the crypt regions (130). This is the level where most IgA^+^ PCs occur also in the human gut (131), and scattered sIgA^+^ memory cells with a proliferative potential are present in human intestinal lamina propria (132). Also, local clonal expansion of precursors for IgA PCs has been reported on the basis of detected Ig_HV_ sequences in human ileal mucosa (133), but the dependency on ILFs is difficult to evaluate in such experiments (113). Thus, rectal immunization elicits particularly high levels of IgA antibodies in colorectal secretions and feces both in experimental animals and humans, apparently reflecting enhanced stimulation by combined exposure of ILFs and lamina propria to the same antigen (34).

Human intestinal IgA^+^ plasmablasts/PCs are special in that they express surface IgA with a functional BCR and therefore may be modulated *in situ* by antigens with potential implications for their survival and regulation (134, 135). It has been suggested that the half-life of PCs varies from a few days to several months, and those ending up in the gut may be particularly short-lived (35). Notably, however, results obtained with isolated PCs may be misleading (108). Thus, at least a fraction of human duodenal PCs are quite long-lived *in vitro*, but only when cultured in whole tissue fragments (136), in agreement with the above cited mouse experiments showing IgA^+^ PC persistence depending on lamina propria stromal cells.

A proliferation-inducing ligand expressed outside of MALT structures may contribute to the survival of lamina propria PCs similarly to the role of BAFF in the bone marrow (137). In addition, the impact of luminal antigens on the conventional B2 cell-dependent effector arm of the SIgA system is most likely mediated largely via “second signals” from activated CD4^+^ T cells. Compartmentalized variables in this process could be a high density of MHC class II molecules (138), allowing only trace amounts of foreign antigens or anti-idiotypic antibodies to elicit sufficient second signals for B cells. Interestingly, in human salivary and lactating mammary glands where there is little exposure to exogenous antigens, IgA^+^ PCs accumulate preferentially adjacent to HLA-DR-expressing epithelial ducts (139, 140), while serous-type acinar cells produce CCL28 which attracts IgA^+^CCR10^+^ plasmablasts (141). Nevertheless, the density of PCs at various secretory effector site reflects topical availability of exogenous antigens, which is high in the gut and lacrimal glands (Figure 7) – the latter secretory site being connected to the heavily antigen-exposed conjunctiva with several small ducts.

## Specific and Cross-Reactive Antibodies to Commensals

In the germ-free appendix model in rabbits, it was shown that only selected commensal bacteria are really efficient in promoting GALT development, and that this ability depended on certain stress responses in the same bacteria, suggesting a non-specific impact on GALT (142). Another study concluded that commensal bacteria could promote the GC reaction in PPs and MLNs by interacting with innate immune receptors, thus being independent of BCR engagement (143). These experiments help to explain how the gut microbiota might drive production of large amounts of IgA with a restricted BCR repertoire and the capacity to bind with low affinity to redundant epitopes of commensal bacteria (144).

### T cell-dependent and -independent responses

Microbial substances called Type 1 T cell-independent (TI-1) antigens are directly mitogenic for B cells – including sugars, lipid structures, and certain nucleic acids. Type 2 T cell-independent (TI-2) antigens, on the other hand, are not by themselves mitogenic but cause extensive cross-lining of BCR by repeating epitopes; their B-cell activation is exerted through synergy with soluble factors (e.g., cytokines) and interaction with various types of accessory cells. There are notable species differences in the mechanisms involved; for instance, while LPS acts as a TI-1 antigen on mouse B cells, it acts as a TI-2 antigen on human B cells. Interestingly, microbial polysaccharides may also function as T cell-dependent antigens and exert profound homeostatic effects on the immune system by stimulating CD4^+^ T cells after being presented by DCs on MHC class II molecules. Thus, it has been shown in germ-free mice monocolonized with the ubiquitous gut commensal *B. fragilis*, that a single bacterial polysaccharide may exert a striking impact both on lymphoid organogenesis and immune modulation (145).

It is well established that the gut microbiota is critically required for activation of GALT with normal intestinal PC development (146), although food proteins also are important for the maturation of the mucosal immune system (147). There is increasingly awareness of the interdependence of diet, commensal bacteria, and immunity (148). Interestingly, because intestinal commensals apparently contribute quite substantially to the shaping of the BCR repertoire of the host, the speculation has been raised that perhaps the GC reaction originated evolutionary in GALT to generate a protective antibody repertoire that was not antigen-specific but rather cross-reactive (149).

The indigenous microbiota might in this context act as polyclonal B-cell activators through several mechanisms, including TLR signaling (150). Experimental studies have attempted to dissect mechanistically this concept by analyzing the intestinal immune response in germ-free mice after monoassociation with a variety of non-invasive, commensal bacteria (151, 152). It was generally found that these microbes induced a GC reaction in GALT with generation of IgA^+^ plasmablasts/PCs which accumulated in the lamina propria and produced both “natural” polyreactive (or cross-reactive) and specific IgA. Individual bacterial species were shown to differ, however, with regard to the maximal amount of total IgA induced and the fraction that could be shown to be specific for antigens of the colonizer (153). All tested bacteria apparently elicited a waxing followed by a long-term waning IgA response, which was accompanied by a GC reaction that notably showed a much more rapid development as well as decline. This could be attributed to the “shielding” of GALT from microbial antigens by the production of specific SIgA antibodies, because of the relatively long-term persistence of both specific and “natural” IgA-producing PCs in the lamina propria.

Such homeostatic immune modulation has been particularly well documented with segmented filamentous bacteria (SFB, related to *Clostridia*), which become a major gut colonizer of the distal ileum of mice after weaning. Colonization of formerly germ-free weanlings resulted in a transient GC reaction in GALT and seeding of the lamina propria with IgA^+^ plasmablasts, providing an SIgA level comprising 50–70% of that seen in conventional mice; but notably, only about 1% of this IgA showed specificity for the SFB (154, 155).

### Immunological memory and homeostasis

Several uncertainties need to be solved before full understanding of the biology of the mucosal IgA system is achieved, and one of the open questions concerns the specificity of the intestinal immune response (113). Monoassociation of germ-free mice with SFB, followed by super-colonization with *Morganella morganii* after 100 days, induced little change in production of total intestinal IgA although the specific response to *M. morganii* increased 20-fold compared to that against SFB (154). The chronic GC reaction observed in GALT of conventional mice is therefore most likely caused by continuous exposure of the gut to novel microbial antigens. Thus, germ-free mice monoassociated with the comensal *B. thetaiotaomicron* showed that specific SIgA antibodies directed against a single epitope (capsular polysaccharide A, PSA) inhibited activation of innate response markers such as oxidative burst and NFκB, thereby inducing crucial modulation of immune homeostasis in the gut as well as microbial antigenic drift (156).

Furthermore, when germ-free mice were colonized with an *E. coli* mutant, and then exposed to other commensal gut bacteria, the memory IgA response to *E. coli* was dampened by attrition, suggesting adaptation of secretory immunity to the predominant microbial epitopes (157). Thus, the sustained colonization of gut bacteria exhibiting novel epitopes may provide the necessary chronic bystander stimulation of previously induced cross-reactive and specific IgA production. SIgA antibodies can hence control the intestinal microbiota in a non-inflammatory, symbiotic, or mutualistic relationship with the host (158, 159).

Notably, at least one fourth of the IgA^+^ PCs in human ileal lamina propria have been shown to produce polyreactive antibodies, which nevertheless were found to be somatically mutated with signs of antigen-driven selection (160). Other studies have shown that IgA autoantibodies produced in duodenal mucosa of patients with celiac disease are of high affinity but with little adaptation by SHMs, exhibiting mainly a germline repertoire (161). In this context it is of considerable interest that mouse experiments recently indicated the presence of two disparate differentiation pathways for memory B cells – one dedicated to generation of high-affinity somatic antibody mutants, and the other with preserved germline specificities to arm the host for rapid responses to encountered variants of potentially dangerous antigens (162) – perhaps including commensals with the potential of causing disease, so-called pathobionts (159, 163).

The near-germline feature is characteristic for the IgA repertoire of human neonates (164), as also reflected in neonatal secretions (165). This situation is followed postnatally by a slow immune maturation with a SHM frequency of IgA V_H_-gene transcripts up to 25% of the adult level at around 5 months of age (164). Both the duodenal and the parotid PC frequency of IgA V_H_ mutations of adults is much higher than that in adult human spleen, apparently because of the constant antigenic pressure on MALT (166). In clean laboratory rats, however, a more restricted IgA repertoire (near germline) was revealed in salivary glands than in the distal small intestine, probably reflecting the regional difference in bacterial load (167).

## How Can Secretory Antibodies Discriminate Between Commensals and Pathogens?

Classical immunological B-cell memory depends on T cell-dependent and long-lived clones which have proliferated in GCs after antigen recognition on FDCs, with further maturation of BCR affinity by point mutations in the V(D)J exons of Ig H and Ig light (L) chains. This process of SHM may be enhanced when antigen recall drives B cell to re-enter the dark zone for a new GC cycle (Figure 6), or to enter a preformed follicle after exit from the GC (168). Attempts have been made to characterize murine memory B cells by their transcriptional program in relation to Ig isotype (169) as both SHM and class-switch recombination (CSR) depends on activation-induced cytidine deaminase (AID) which targets specific DNA motifs in the B cell (170, 171). However, characterization of human memory B cells has mainly been based on phenotyping and the molecular history of SHM and proliferation (172). CD20^+^CD27^+^ B cells have been taken to represent memory cells and their expansion in children between 4 and 18 months of age was found to be related to gut colonization with *E. coli* and bifidobacteria (173).

With regard to circulating IgA^+^ B cells, the CD27-expressing subset was indeed considered the best memory candidate with signs of a prolonged AID impact during multiple immune responses, most likely involving MALT (172). However, the same authors also reported the presence of a minor subset of blood CD27^−^IgA^+^ B cells which seemingly were completely T cell-independent and showed molecular similarities to IgA^+^ B cells in colonic mucosa. This finding was deemed to support the notion that IgA2-producing PCs in the distal human gut develop in the lamina propria by direct CSR from IgM to IgA2 in a T cell-independent manner (80). As alluded to previously, this view has been refuted by our recent molecular study of IgA-subclass switching in intestinal effector sites compared with inductive sites (Lin et al., in revision).

Although mucosal IgA responses to commensal bacteria may be multi-centered, of low affinity and diverse, oral immunization of mice with a cholera toxin-adjuvanted novel antigen was recently shown to result in a strongly oligoclonal response of affinity-matured IgA^+^ B cells (174). Interestingly, it was found that the response was highly synchronized throughout the entire intestine by involving multiple PPs. Thus, by reutelizing already existing CGs, antigen-specific B cells would be subjected to clonal expansion and SHM, probably in the manner discussed previously. This process was shown to require antigen recall by multiple immunizations, and the study helps to clarify mechanisms underlying the functional flexibility of mucosal anti-microbial IgA responses: from “natural” polyreactive (or cross-reactive) low affinity to a specific high-affinity “classical” response – a distinction that is a major challenge to mucosal vaccine design (113).

**Figure 8:**
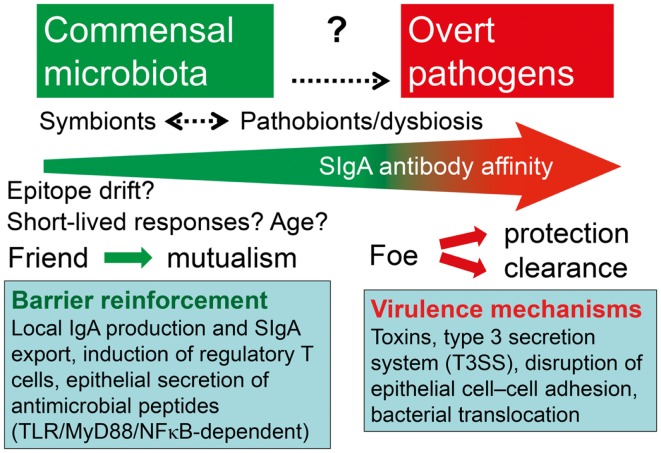
**Hypothetical depiction of how the intestinal immune system handles symbionts and potentially pathogenic residents (pathobionts) of the commensal microbiota versus overt exogenous pathogens**. Secretory IgA (SIgA) antibody levels against commensal bacteria may go in waves because of epitope drift and shielding of gut-associated lymphoid tissue from antigen uptake. The overall affinity of SIgA antibodies probably increases with age and may be enhanced or reduced against pathobionts during dysbiosis, and particularly raised by persistent stimulation with overt pathogens. One goal of mutualism with commensals is mucosal barrier reinforcement by mechanisms listed such as SIgA export and induction of regulatory T cells, whereas pathogens exhibit various virulence mechanisms to break the barrier.

The difficult issue of affinity maturation of mucosal B cells and how the mucosal immune system can distinguish between the indigenous microbiota and overt exogenous pathogens has been discussed in several recent articles (175–177). The mucosal barrier and its reinforcement by SIgA, as well as the mucosal immunoregulatory network, require both adaptive and innate induction by antigens and conserved microbe-associated molecular patterns (MAMPs) – the latter activating germline-encoded cellular PRRs such as TLRs (178, 179). It is elusive how such receptors would be able to discriminate between signals provided by MAMPs from commensals and MAMPs from pathogens (previously called pathogen-associated molecular patterns, PAMPs); but this distinction is clearly required to elicit tolerogenic versus proinflammatory immune responses needed for protection against invasive infections (Figure 8). Various scenarios may be visualized – the most likely being that overt pathogens, in addition to signaling through PRRs and BCRs, exhibit special danger signals, or immune evasion mechanisms related to the pathogenicity – that is, factors determining virulence and invasiveness (180–182), or so-called effector-triggered immunity (183, 184), while competition for metabolically shared nutrients may also be involved (159, 185, 186).

## What Does Antibody Coating of Commensals Mean?

**Figure 9:**
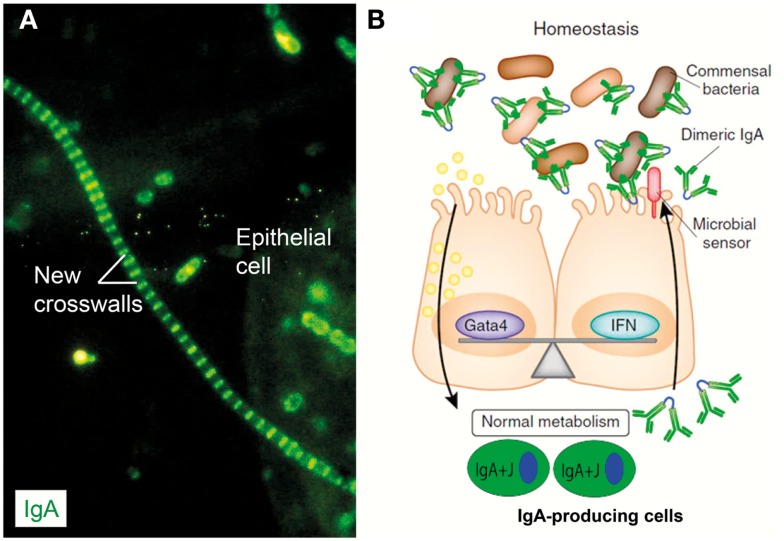
**IgA coating of commensal bacteria may modulate mucosal immunity and metabolism**. **(A)** Direct immunofluorescence staining of bacterial sediment from whole saliva to demonstrate *in vivo* IgA coating. Contaminating epithelial cell is faintly visualized because of autofluorescence. Numerous cocci (mainly diplococci) – partly adhering to epithelial cell – have bound IgA, which also decorates older cell-wall segments of streptococci forming long chains, whereas new crosswalls formed by growth *in vitro* after sampling are negative as indicated. Adapted from Brandtzaeg et al. (57) (Original magnification: ×2000). (**B)** In healthy individuals, IgA^+^ mucosal plasma cells produce dimers with J chain (IgA + J) which are exported to the epithelial surface by the polymeric Ig receptor (see Figure 1). Secretory IgA binds to commensal bacteria, and the IgA-coated bacteria modulate mucosal immunity and homeostasis by delivering signals through innate microbial sensors (red) such as pattern recognition receptors on the epithelial cells. In addition to enhancing innate defense through immune pathways controlled by interferon (IFN) cytokines, these signals also regulate the intake of food lipids through metabolic pathways controlled by the transcription factor Gata4. When IgA is lacking (not shown), the gut epithelium upregulates its expression of IFN-dependent innate defense genes to compensate for the lack of adaptive IgA immunity. This upregulation leads to a downregulation of the genes controlled by Gata4 (196). The resulting gene imbalance impairs the epithelial absorption of lipids, such as cholesterol, resulting in metabolic disorders with reduced leptin levels and fat storage. Modified from Chorny and Cerutti (197).

*In vivo* coating of bacteria with IgA present in external secretions can be directly demonstrated by immunostaining (Figure 9A); and although this apparently does not inhibit bacterial growth (57), it is considered to provide containment of the microbiota, counteract invasiveness, and contribute to immune homeostasis (24, 51).

In human feces, some 40% of the anaerobic bacteria are normally coated with IgA (187) and this phenomenon can be observed in early childhood (188). Such IgA containment of commensals, without eliminating them, is probably important for the mutual host–microbe interaction, contributing to sustainable homeostasis by dampening proinflammatory signaling in the host and providing an immune pressure on commensal bacteria (159, 189). This results in antigenic drift without altered composition of the microbiota, or so-called dysbiosis (156, 190). Most likely this IgA coat largely represents “natural” cross-reactive antibodies (116, 191) but may also depend on innate properties of SIgA, as discussed previously. In mouse experiments with *Salmonella typhimurium* infection it was found that bacteria coated with such “natural” IgA showed reduced shedding and less liability to horizontal spreading by the fecal-oral route (192).

In mice, the IgA coating of gut bacteria has been shown to be unrelated to the total amount of SIgA exported to the intestinal lumen, suggesting that a specific reaction is involved (193). Also, other recent mouse experiments demonstrated that the commensal coating with IgA in feces depended on appropriate clonal B-cell selection and affinity maturation in GCs of GALT, and perhaps to some extent also in the lamina propria (190). Thus, this finding showed that the coating to a substantial degree reflects a specific IgA response. It has therefore been speculated that pathobionts might show increased IgA coating in the gut lumen. This idea is supported by findings in patients with inflammatory bowel disease, where there is dysbiosis (159, 194) and the fraction on anaerobic bacteria with IgA is raised to 65%, with 45% also carrying IgG (195). Thus, increased antibody coating could reflect dysbiosis (Figure 8). However, in celiac disease, where there is also dysbiosis, the IgA coating of fecal bacteria is significantly reduced (188). So the biological significance of this phenomenon remains uncertain, and the degree of IgA coating might reflect a combination of innate and specific bacterial binding properties of SIgA.

Altogether, SIgA does not seem to cause clearance of commensal bacteria, but controls in various ways their colonization and inhibits the penetration of agents that could potentially cause hypersensitivity reactions or infection (24). In the absence of B cells, or when IgA is lacking, the intestinal epithelium of mice will, in response to commensal bacteria, upregulate in an NFκB- and interferon-dependent manner its innate defense (24, 189), and this could be at the expense of expression of genes that regulate fat and carbohydrate metabolism (196). As a consequence, the epithelial gene signature might correlate with the development of lipid malabsorption (197). The intestinal epithelial barrier is a cross-road between surface defense and nutrition, and SIgA is apparently essential to keep the balance between these two functions and thus maintain mucosal homeostasis (Figure 9B). Lack of SIgA probably explains that patients with common variable immunodeficiency may have a similar gene expression profile in their duodenal mucosa and show malabsorption with fatty stools (196) – despite frequent surface defense compensation with increased intraepithelial lymphocytes, particularly the CD8^+^ subset (198).

## Concluding Remarks

Secretory immunoglobulin A constitutes the largest humoral immune system of the body and performs antigen exclusion at mucosal surfaces and neutralizes virus and endotoxin within epithelial cells without causing tissue damage (24, 199). The enormous innate drive of the mucosal immune system does not only enhance anybody diversity but also immunological memory. Mainly by its extensive polyreactivity, innate-like SIgA is persistently containing commensal bacteria outside the epithelial barrier; but by increased antibody affinity SIgA can also target invasion of pathogens and penetration of harmful antigens after mucosal infection or vaccination. Host resistance to toxin-producing bacteria such as *V. cholera* and enterotoxigenic *E. coli* seems to depend largely on SIgA, and so does herd protection against horizontal fecal-oral spread of enteric pathogens under naïve or immunized conditions – with a substantial innate impact both on cross-reactivity and memory.

Like natural infections, live attenuated vaccines or adequate combinations of non-replicating vaccines and mucosal adjuvants administered by the oral route to target GALT, give rise not only to SIgA antibodies but also to longstanding serum IgG and IgA responses (199). However, there is considerably disparity with regard to migration of memory/effector B cells from mucosal inductive sites to secretory effector sites and systemic immune organs. Also, although immunological memory is generated after mucosal priming, this may be masked by a self-limiting response shielding the inductive lymphoid tissue in the gut. The intranasal route of vaccine application targeting NALT may be more advantageous for certain infections, but only if successful stimulation is achieved without the use of toxic adjuvants that might reach the central nervous system (200). The degree of protection obtained after mucosal vaccination ranges from reduction of symptoms to complete inhibition of re-infection. In this scenario, it is often difficult to determine the relative importance of SIgA versus serum antibodies, but infection models in knockout mice strongly support the notion that SIgA exerts a decisive role in protection and cross-protection against a variety of infectious agents.

Nevertheless, relatively few mucosal vaccines have been approved for human use, and more basic work is needed in vaccine and adjuvant design, including particulate or live-vectored combinations (200). It is a fundamental problem in this field that clinical trials are risky and expensive, so much of the basic work has to be performed in experimental animals such as mice. There are always difficulties in knowing to what extent immunological information obtained in animals can be directly transferred to human application. Thus, considerable species differences exist in subsets of APCs and also with regard to the SIgA system. For instance, human hepatocytes do not express pIgR whereas rodent hepatocytes do (17). As a consequence, a large proportion of SIgA in the lumen of the upper small intestine of these animals originates from peripheral blood because the liver continuously “pumps” circulating pIgA into bile after its complexing with SC. It is therefore difficult to know to what extent SIgA antibodies in rodent gut fluid reflect a local mucosal immune response. Ultimately, though, the great efforts that are currently invested in developing novel mucosal vaccines and adjuvants will hopefully enhance the possibilities to exploit the SIgA system in mankind’s fight against infectious diseases, and thereby improving global health (200).

## Conflict of Interest Statement

The authors declare that the research was conducted in the absence of any commercial or financial relationships that could be construed as a potential conflict of interest.
